# Monthly Summary

**Published:** 1877-09

**Authors:** 


### MONTHLY SUMMARY.

---

*The Cold Bath in Infantile Diarrhœa.*—This has long been known as a most efficacious measure, even in extremis. An Italian practitioner, Dr. Wocke, has lately been drawing attention to the same plan of treatment. He refers more particularly to the terrible epidemics of diarrhœa which prevail in summer. The epidemic is due, he says, in part to the deleterious influence of the elevated temperature on the infantile organism, and in part to the injurious effect which the heat exerts on the aliment, the milk, and the air inspired. For this state of things he recommends cold bathing, of which he has a high opinion. Wasting children, reduced by vomiting and diarrhœa, were as if regenerated by the second day after the baths were commenced. The restlessness disappeared; sleep was restored, the appetite increased, and the diarrhœa diminished. The cold baths act on the child as a tonic, and internal remedies then exert a better influence. Dr. Wocke commences his treatment with cold douches to the head and stomach; then passes to baths, commencing at a temperature of 26° C., and reducing them to 22.° Three baths a day are sufficient.—*Med. and Surg. Reporter.*

---

*Carbolic Acid in Diarrhœa*—A correspondent, Dr. S. W. Palmer, of Ohio, writes us;—Recently, I have employed carbolic acid in the treatment of diarrhœa, both acute and chronic form of the disease, with signal success. It is given in one or two drop doses, largely diluted with water, from two to four hours apart. It controls pain, and corrects the fetor of the discharges, and otherwise cures the disease. If severe pain be present, and the discharges profuse, opium and creta preparata, with astringents, may be alternated with it. But my experience is that carbolic acid in most cases is all that is required.—*Med. and Surg. Reporter.*

---

*Croton Chloral in Dentistry.*—Dr. C. I. Cleborne (*Medical Journal*) uses croton chloral as an internal and local anæsthetic in toothache. He has derived prompt relief from its use in 5-10 grain doses in the toothache of dyspepsia and pregnancy, and in those aggravating cases which occur in rheumatic and neuralgic patients. Failing to relieve by the ordinary remedies a severe attack of toothache, due to dental caries, I found that a mixture of equal parts of crystallized carbolic acid, croton chloral hydrate and solid Japanese oil of peppermint promptly removed the pain and obtunded all sensitiveness during the period of filling. In filling teeth, when it is not necessary nor expedient to destroy the pulp by arsenic or other escharotic, nearly all sensitiveness may be allayed, during the preparation of the cavity, by the following plan: Arm a fine brooch with a small ball of cotton wool, dip it into the mixture, removing any superfluous material by a little pressure: introduce it into the dental cavity and retain in position by the introduction of a small piece of wool. In the course of five or ten minutes the wool may be removed, and the excavation of the cavity may be proceeded with until it again becomes sensitive, when the application should be renewed, and repeated as often as it may be necessary until the cavity is ready for the filling ; a result which may be accomplished with but little or no pain, and without injury to, or the destruction of the nerve.—*Journal of Materia Medica.*

---

*Use of Tobacco by Students.*—In the prospectus of the Berkeley Gymnasium, a preparatory school to the University of California, we find the following sect on on the use of tobacco. Imagine a "young gentleman" sitting down in the smoking room, with a cigar in his mouth, elated with the dignity of his position, and proud of the concession which his instructors have made to his desires, and at the same time contemplating this permissive act, particularly that part of it relating to the "clean and the unclean."

"It is a fact not to be overlooked that a great majority of our boys in California are given more or less to the use of tobacco. We do not defend this or any other species of intemperance. We believe it is destructive to both brain and body. Yet we deem it wise to deal with the matter as an *existing thing*, which it is. We do not believe that it is wholesome for the good morals of a young man to be sneaking around the buildings in quest of any opportunity that may be presented to absent himself from school grounds to practice in secret places or vicious companionship what he would be either afraid or ashamed to acknowledge to his best friends.

We shall therefore have a smoking room comfortably fitted up for the convenience of those students who are addicted to this habit ; *but in all cases we must first have the written authority of parents or guardian.* Under no circumstances will a student be permitted to smoke more than once a day ; namely, during the half hour immediately following dinner. It is our design in this to separate the clean from the unclean, and not allow the influence of tobacco smokers to be felt promiscuously upon young and susceptible pupils, as we are confident is often the case."—*Pacific Med. and Surg. Journal.*

---

*Death from Anæsthetics.*—A death recently occurred at the East Suffolk Hospital, (London,) during the administration of bichloride of methylene and ether for the removal of diseased bone from the leg of a patient, aged fifty-six, who was suffering from syphilitic caries. Death occurred during a convulsion. For some unexplained reason, the coroner forbade a post-mortem, and consequently the immediate cause of death was not ascertained.—*Med. Record.*

*Polypus Growing from the Septum of the Nose.*—A girl aged 15, had been troubled with obstruction of the left nostril and epistaxis for four months. This was found to be due to a pedunculated polypus growing from the septum. In January it was removed under chloroform, and now there is no sign of disease. Polypus growing from the septum is very rare; this one measured over half an inch by one third: it was very vascular, and ulcerated on the surface. Structurally, it consisted of a large number of vessels and adenoid tissue, with many lymph cells.—*British Med. Journal.*

---

*India Rubber Cloth in Diseases of the Skin.*—(*Virginia Clinical Record.*)—M. Hardy, of Paris, has now employed india-rubber cloth instead of poultices or local baths since 1868. It is composed of a layer of caoutchouc adherent to a piece of cotton, and forms an impermeable tissue. After a short time an abundant sweating takes place from the part enveloped, under the influence of which crusts and scales are loosened, the epidermis spreads over the ulcers, and the skin becomes softened. A very rapid modification of the skin takes place; two or three days of application suffice to completely clean a scalp covered with abundant scales of eczema, etc. After forty-eight hours, hands affected with chronic eczema, with fissures and cracks in all directions, become cicatrized, and the skin recovers its suppleness. The treatment is of particular value in the second stage of eczema. Oiled silk cannot take the place of the rubber cloth. This method is also serviceable in psoriasis and chronic lichen.—*Journal of Materia Medica.*

---

*Apthœ.*—Cupri sulphus is an excellent old-fashioned application in the severe forms of cancrum oris, apthous ulceration and gangreneous affections of the mouth. Symonds used five grains finely powdered and thoroughly incorporated in half an ounce of honey. It has also been applied in substance.—*Virginia Med. Monthly.*

---

*Salicylic Acid Bad for Teeth.*—M. Blandeau, of Paris, states that according to dentists, this agent has injurious effects of the teeth. English observers have noticed its effect on the bones, necrosis of the tibia has been assigned to its use. It evidently possesses considerable affinity for the calcareous salts of bone, and we see the urine loaded with lime salts in an ultra-physiological proportion, from the internal use of the acid. The salicylate of soda presents the same danger; and too much caution cannot be taken in the use of any salicylic preparation. —*Drug. Cir. and Chem. Gaz.*

*Acidity of the Gastric Juice of Man.*—Dr. Charles Richet read a paper before the French Academy Sciences recently, in which he treated of the acidity of the gastric juice in man. For some time Prof. Verneuil had a young patient under his care, who labored under the strange affection of having his œsophagus, or upper extremity of the alimentary canal, so contracted as to render the passage not only of food or drink, but even of an instrument quite impossible. The professor had recourse to gastrotomy, an operation until then considered mortal, but which in this case succeeded perfectly, An incision made in the stomach, and kept open artificially, has been transformed into a regular fistula, through which food and drink are administered, and in this state the young man not only lives, but is a useful servant to the hospital. Dr. Richet, having devoted particular attention to this subject, and having examined the workings of the stomach day by day, through the fistula in question, lately communicated the results obtained to the academy. They are as follows : [1.] The average acidity of gastric juice, whether pure or mixed with food, is equal to 1·7 grammes of hydrochloric acid per 1,000 of liquid. It has never been observed to be lower than 0-5 or higher than 32. [2.] The quantity of liquid contained in the stomach exercises no influences on its acidity, which remains nearly invariable, whether the stomach be empty or filled with aliment. [3.] Wine and alcohol increases the acidity of the stomach ; cane sugar diminishes it. [4.] If acid or alkaline liquors be injected, the gastric ones tend rapidly to resume their normal acidity, so that about an hour after the injection the stomach has regained its average acidity. [5.] The gastric juice is more acid during digestion than before or after. [6.] There is a slight increase of acidity toward the end of digestion. [7.] The sensations of hunger and thirst do not depend either on the state of acidity or that of vacuity of the stomach. Such are the results as to acidity, but there are a few others of some interest. Thus fecula, fat and meat stay in the stomach for three or four hours, milk is digested in the course of an hour and a half ; water and alcohol are absorbed much faster, in the course of from 35 to 45 minutes. Food is not transmitted to the pylorus successively, but all in a block.—*Druggists Circular.*

---

*Lacto-Phosphate of Lime as a Tooth Filling.*—The treatment of exposed dental pulps and sensitive dentine is the subject of an interesting paper by Junius E. Cravens, D. D, S. The lacto-phosphate of lime is applied to the exposed pulp, which is carefully sealed and left undisturbed for several weeks. On removing the coverings a new bone is found, its surface continuous with that of the formerly soft dentine, and the sensibility being even below the normal degree. Oxy-chloride of zinc and other substances, however, may produce like results.—*Med. Record.*

*Therapeutic Use of Cold.*—Cold may be employed in the form of ice, or ice poultices (made by mixing bits of ice with a linseed poultice,) or evaporating lotions, or irrigation. Cold acts through the vaso-motor nerves upon the capillaries, constringing and even emptying them of blood ; and this not merely in those capillaries immediately in contact, but owing to the dispersion of the nervous filaments, even at a considerable distance and thus renders the advent of congestion and inflammation physically difficult or even impossible. It should be used with some caution, as, if its action be kept up too long, it may destroy the vitality of a part, as happened lately in a case of femoral hernia, when after operating, I applied ice with a view to prevent peritonitis: this it achieved, but produced a considerable slough of the integuments, which was a long time healing over. The value of cold is typically seen in cases of sprain and of violent wrenchings of a joint. For example, after breaking down adhesions in cases of fibrous anchylosis, I know nothing so certain to obviate the tendency to synovitis as the immediate and continuous application of ice to the roughly handled joint. In the same way, thin bandages wrung out in cold water, kept cold, and applied with a fair amount of pressure, constitute the best of all treatments in cases of recent sprains and contusions. Besides causing contraction of the capillaries, cold, still acting through the trophic nerves, produces muscular contraction, to which property is due its efficacy in causing contraction of the relaxed parturient uterus ; while its former property gives its value as a hæmostatic in cases of oozing from a stump or other parts after operation.—*Druggists Circular.*

---

*Osseous Lesions in Infantile Syphilis.*—(M. Parrot, *Archives de Physilogic*, *Lancet*, Nov., 1876.)—They are the most common manifestation of syphilis, and make their appearance some days, weeks or months after birth. They are diagnosticated from rachitic changes by their appearance in the earlier periods of infancy, and on the other hand, the latter lesions are rarely seen before the sixth month of extra-uterine life. The long bones of the limbs, scapula, ilium and cranial bones are those most frequently affected with the syphilitic taint, while the ribs, clavicles, metacarpal and metatarsal bones and the vertebræ more rarely suffer. The bones are always attacked symmetrically. The lesions are arranged by M. Parrot under four heads, according to the degree of osseous change. Those of the first degree are seen in the fœtus and in infants who live but a few days. The affected bones are covered with a layer of osteophytic growth and the epiphysial cartilages are thickened, so that sometimes the diameter of the bones attains double the normal size. In the 2nd degree, the layers of new bone are not so compact and are deposited usually upon the lower extremities of the humerus, the upper of the radius, the anterior surface of the femur, and the inner surface of the tibia. At the same time a gelatiniform atrophy of the cancellous tissue takes place which soon extends to the compact substance and lastly to the layer subjacent to the epiphysial cartilage. This osseous degeneration gives rise to the syphilitic pseudo paralysis of the new-born. In the 3rd degree, which affects older infants, an hypertrophy of the medullary substance gradually encroaches upon and displaces the osteophytic productions of an earlier date. This occurs most frequently in the lower third of humerus. In the 4th degree, spongy tissue develops itself at the periphery and the extremities of the long bones, which lesion occurring after the sixth month of life may pass into and be mistaken for those of rachitis.—*Detroit Med. Journal.*

---

*Dangerous Prescriptions.*—Some cases are mentioned in our exchanges in which corrosive sublimate has been dispensed for calomel in consequence of either prescriber or dispenser being unable to follow the changes which have been made in the nomenclature of these two chlorides. We have always doubted the propriety of a pharmacopœia attempting to follow the shifting views of chemical theory. A name for a drug need not be chemically correct. A worse case is reported in which *hyd. chlor.* was written by a physician who intended it for hydrate of chloral. Corrosive sublimate was dispensed, and the patient nearly killed, life being saved by vomiting occuring immediately on swallowing the poison, and timely aid. A critic who pronounces the physician's act a blunder, and the dispenser's worse, says the rule should be religiously observed never to abbreviate those words but to write them in full *hydratis chlorali*, or else put it in English. Now the word *chloral* is not declinable in Latin, and should, moreover, precede *hydratis*. Its proper position would render such another blunder less likely, and should therefore be assigned to it, and we hope critics will not generally violate grammar by adding terminations to undeclinable nouns.—*The Doctor.*

---

Dr. Edward Warren, (Bey,) formerly of Baltimore, a prominent American physician of Paris, has just been created a Knight of the Order of Isabel the Catholic, as a recognition of the professional skill displayed by him in the successful treatment of some Spanish personages of high position.

